# Next-generation sequencing profiling of mitochondrial genomes in gout

**DOI:** 10.1186/s13075-018-1637-5

**Published:** 2018-07-06

**Authors:** Chia-Chun Tseng, Chung-Jen Chen, Jeng-Hsien Yen, Hsi-Yuan Huang, Jan-Gowth Chang, Shun-Jen Chang, Wei-Ting Liao

**Affiliations:** 1 Department of Internal Medicine, Kaohsiung Municipal Ta-Tung Hospital, Kaohsiung, Taiwan; 2 Department of Internal Medicine, Kaohsiung Medical University Hospital, Kaohsiung, Taiwan; 3 Department of Internal Medicine, College of Medicine, Kaohsiung Medical University, Kaohsiung, Taiwan; 4 Division of Rheumatology, Department of Internal Medicine, Kaohsiung Medical University Hospital, Kaohsiung, Taiwan; 5 Graduate Institute of Clinical Medicine, College of Medicine, Kaohsiung Medical University, Kaohsiung, Taiwan; 6 Department of Laboratory Medicine and Epigenome Research Center, China Medical University Hospital, China Medical University, Taichung, Taiwan; 7 Department of Kinesiology, Health and Leisure Studies, National University of Kaohsiung, Kaohsiung, Taiwan; 8 Department of Biotechnology, College of Life Science, Kaohsiung Medical University, Kaohsiung, Taiwan; 9 Department of Medical Research, Kaohsiung Medical University Hospital, Kaohsiung, Taiwan

**Keywords:** Mitochondria, Gout, Next-generation sequencing

## Abstract

**Background:**

Accumulating evidence implicates mitochondrial DNA (mtDNA) alleles, which are independent of the nuclear genome, in disease, especially in human metabolic diseases. However, this area of investigation has lagged behind in researching the nuclear alleles in complex traits, for example, in gout.

**Methods:**

Next-generation sequencing was utilized to investigate the relationship between mtDNA alleles and phenotypic variations in 52 male patients with gout and 104 age-matched male non-gout controls from the Taiwan Biobank whole-genome sequencing samples. Differences from a reference sequence (GRCh38) were identified. The sequence kernel association test (SKAT) was applied to identify gout-associated alleles in mitochondrial genes. The tools Polymorphism Phenotyping, Sorting Intolerant From Tolerant (SIFT), Predict the pathology of Mutations (PMUT), Human Mitochondrial Genome Database (mtDB), Multiple Alignment using Fast Fourier Transform (MAFFT), and Mammalian Mitochondrial tRNA Genes (Mamit-tRNA) were used to evaluate pathogenicity of alleles. Validation of selected alleles by quantitative polymerase chain reaction of single nucleotide polymorphisms (qPCR SNPs) was also performed.

**Results:**

We identified 456 alleles in patients with gout and 640 alleles in non-gout controls with 274 alleles shared by both. Mitochondrial genes were associated with gout, with *MT-CO3*, *MT-TA*, *MT-TC*, and *MT-TT* containing potentially pathogenic gout-associated alleles and displaying evidence of gene-gene interactions. All heteroplasmy levels of potentially pathogenic alleles exceeded metabolic thresholds for pathogenicity. Validation assays confirmed the next-generation sequencing results of selected alleles. Among them, potentially pathogenic *MT-CO3* alleles correlated with high-density lipoprotein (HDL) levels (*P* = 0.034).

**Conclusion:**

This study provided two scientific insights. First, this was the most extensive mitochondrial genomic profiling associated with gout. Second, our results supported the roles of mitochondria in gout and HDL, and this comprehensive analysis framework can be applied to other diseases in which mitochondrial dysfunction has been implicated.

**Electronic supplementary material:**

The online version of this article (10.1186/s13075-018-1637-5) contains supplementary material, which is available to authorized users.

## Background

As the primary energy producers of the cell, mitochondria are critical to cellular metabolism. While metabolism is the function most often associated with mitochondria, the organelle plays a vital role in other cellular processes including inflammation [1], suggesting that mitochondrial dysfunction could have far-reaching effects on human health and diseases. Indeed, past studies have revealed causal roles of mitochondrial DNA (mtDNA) mutations and mitochondrial dysfunction in diseases with altered metabolism and inflammatory responses, for example, atherosclerosis [2]. These findings may apply as well to other metabolic and inflammatory diseases.

Gout is a prototype disease with dysregulated uric acid metabolism and inflammatory response triggered by monosodium urate (MSU) crystals [3]. Multiple lines of evidence suggest that mitochondrial dysfunction is a key element in the pathogenesis and pathophysiology of gout. First, oxidative respiration in hepatic mitochondria decreases urate production [4]. This is further evidenced by the Warburg effect, a metabolic condition characterized by the switch from oxidative phosphorylation, which requires mitochondrial respiration, to aerobic glycolysis, which increases production of uric acid during periods of inflammation [3]. Second, mitochondria constitute the signal-integrating organelle for inflammasome activation, which initiates gout flares [1, 3]. In this frame of reference, we hypothesize that mtDNA variations represent a mechanism that increases uric acid production and/or facilitates inflammasome activation, which are both involved in the development of gout.

In past research, genome-wide association studies (GWAS) were performed to explore a number of nuclear loci associated with various complex diseases, including gout [5]. However, nuclear loci identified in GWAS collectively explain a relatively small proportion of phenotype variance [5]. Novel approaches to identifying genetic loci other than traditional loci, for example, mtDNA, which influence disease risk, prognosis, or the response to treatment are urgently needed. Given that mutant mtDNA alleles usually coexist with wild-type (normal) mtDNA in cells (heteroplasmy) [6], standard genotype calling for nuclear loci may be controversial when applied to mtDNA due to the metabolic threshold effects in the relationship between phenotypes and mutation loads [6, 7]. Therefore, it is recommended to account for heteroplasmy using individual-level allele frequencies obtained from sequencing data rather than genotype calls obtained by algorithms that were designed for nuclear loci [7]. Next-generation sequencing has now been applied for identification of DNA alleles to decipher the genetic bases of complex traits and has been expanded to analyze mtDNA [8]. In order to elucidate the genetic landscape of mitochondria in gout patients and the impact of mitochondria alleles on phenotypes, whole genome sequencing samples from the Taiwan Biobank (TWB) were utilized [9]. mtDNA alleles from 52 patients with gout and from 104 age-matched non-gout controls were analyzed according to their location and distribution between different groups to capture the burden of mitochondria alleles in gout and the association between mitochondrial alleles and clinical phenotypes. This study presents the most extensive mtDNA genotyping relating to gout and may be relevant for other diseases associated with mitochondrial dysfunction as well.

## Methods

### Patient selection

All samples in this study were drawn from the TWB, a cohort established to increase our understanding of the relationships among genetics, environment, and the etiology/progression of disease, including gout [9]. This cohort was based on the recruitment and monitoring from the general Taiwanese population, and has been utilized in previous genetic studies [9]. During recruitment, all patients provided informed consent to undergo genomic sequencing and DNA in peripheral blood leukocytes was extracted for genomic sequencing. This study was approved by the Institutional Review Board (TSMHIRB 16–006-CO) and the TWB is governed by the Ethics and Governance Council (EGC) and the Ministry of Health and Welfare in Taiwan. All experiments were performed in accordance with relevant guidelines and regulations.

From the TWB, 52 male patients with self-reported gout who underwent whole-genome sequencing were selected. For the control cohort, 104 male patients who self-reported the absence of gout and underwent whole-genome sequencing were matched by age to our patients with gout and were randomly selected from the TWB. All participants were reported as being of Han Chinese ancestry. Additionally, previous studies utilized a similar method of self-reported gout and suggested that self-reporting of physician-diagnosed gout retained good reliability and sensitivity [10, 11].

### Mitochondrial genomic sequencing

All patients of this study underwent whole-genome sequencing on the Illumina Hiseq or Ion Proton platform. For the Illumina Hiseq platform, mitochondrial DNA sequences were aligned with Isaac version 01.13.10.21. Variant calling was performed with Isaac Variant Caller version 2.0.17, Grouper version 1.4.2, and CNVseg version 2.2.4. Alleles were annotated with ANNOVAR version 2014Jul14. For the Ion Proton platform, mitochondrial DNA sequences were aligned with TMAP version 4.4. Variant calling was performed with torrent variant caller version 4.2.2, and alleles were annotated with Ensembl VEP. The distribution of sequencing platforms was similar in gout and non-gout controls (*P* = 1.000) (Additional file 1). The symbols and full names of the total 37 genes in human mitochondria are listed in Additional file 2. Complete assembly of the mitochondrial genome was obtained in all cases.

### Next-generation sequencing data analysis

**Fig. 1:**
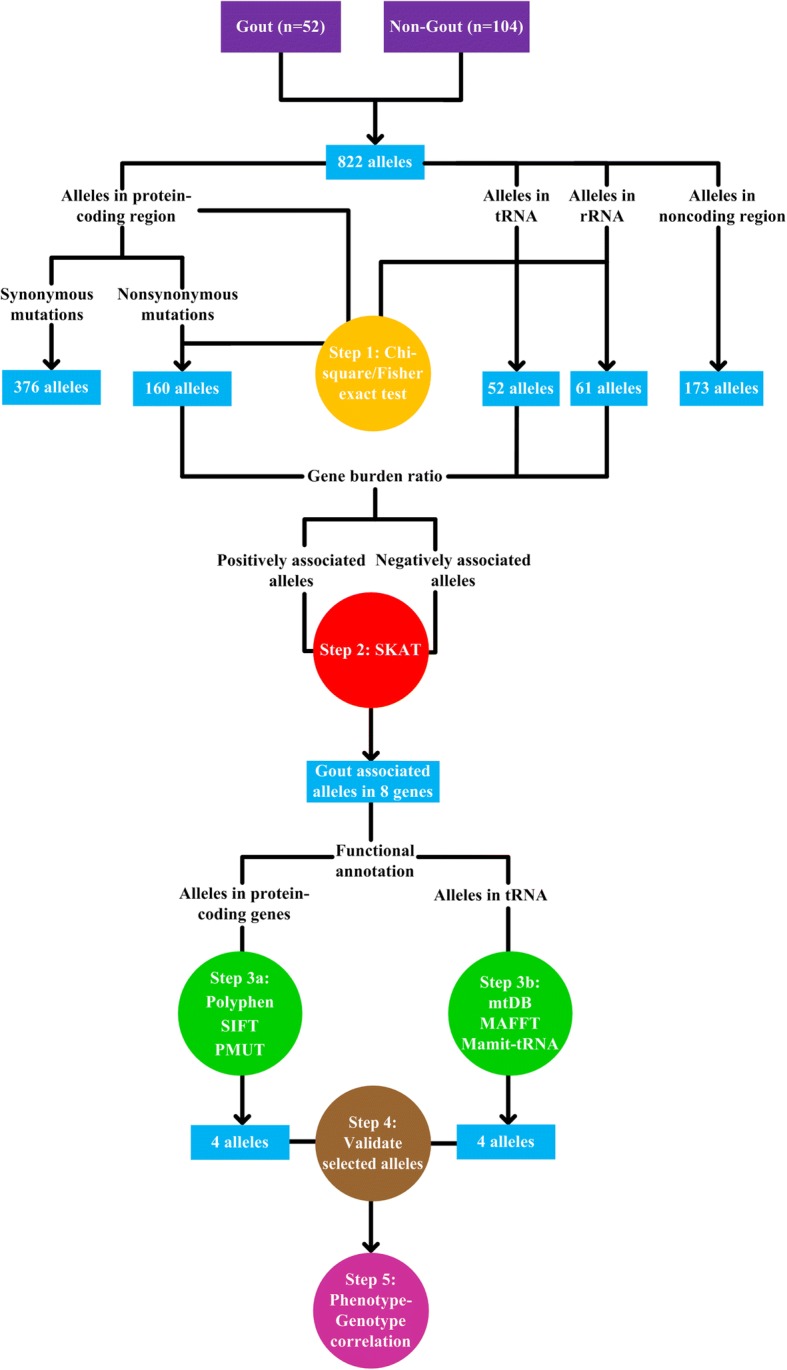
Schematic representation of the next-generation sequencing data analytical workflow. After comparison with the reference sequence (GRCh38), 822 alleles different from the reference sequence (GRCh38) in patients with gout and non-gout controls were recorded (376 synonymous alleles and 160 nonsynonymous alleles in protein-coding genes, 52 alleles in tRNA genes, 61 alleles in ribosomal RNA (rRNA) genes, and 173 alleles in the noncoding region). In the Step 1, the enrichment of alleles in patients with gout and non-gout controls were identified using the chi-square test or exact test, as described in “Methods”. Next, gene burden ratio was calculated to characterize alleles as positively associated alleles or negatively associated alleles for Sequence Kernel Association Test (SKAT) analysis (Step 2). Eight genes associated with gout were obtained from SKAT analysis and annotated with Polymorphism Phenotyping (Polyphen), Sorting Intolerant From Tolerant (SIFT), Predict the pathology of MUTation (PMUT) (for alleles in protein-coding genes) (Step 3a) and the Human Mitochondrial Genome Database (mtDB), Multiple Alignment using Fast Fourier Transform (MAFFT), and Mammalian Mitochondrial tRNA Genes (Mamit-tRNA) (for alleles in transfer RNA (tRNA) genes) (Step 3b). Genotyping results of selected alleles was further validated (Step 4). After functional annotation, four alleles in protein-coding genes and four alleles in tRNA genes were singled out for genotype-phenotype correlations (Step 5)

We first compared sequencing results with the reference sequence (GRCh38), and recorded all mutant alleles that differed from the reference sequence. Mutant alleles were classified into 3 distinct groups: Group 1 (shared by patients with gout and non-gout controls), Group 2 (found in patients with gout only), and Group 3 (found in non-gout controls only). These 3 groups of alleles were further grouped according to genes to which they belonged. We then identified genes with significant enrichment of certain alleles in gout or age-matched non-gout controls (Fig. 1, Step 1) using the chi-square test or exact test, which is in line with the approach adopted in a previous study [12].

### Sequence kernel association test (SKAT) analysis

To explore which genes were associated with gout (Fig. 1, Step 2), gene burden ratio was calculated by dividing the allele frequency in gout by the allele frequency in non-gout controls, and we divided alleles in each gene to positively associated alleles (gene burden ratio > 1) and negatively associated alleles otherwise. SKAT, which applied a multivariate regression model that accommodates both positively associated and negatively associated alleles simultaneously to uncover genes associated with gout and to detect genetic interactions, was performed to clarify the contribution of gout from mitochondrial genes and genetic interactions [13].

### Functional annotation of nonsynonymous alleles in protein-coding genes

After identifying alleles contributing to gout, we proceeded to evaluate the pathogenicity of these alleles (Fig. 1, Step 3a, Step 3b). The potential functional impact of all identified nonsynonymous alleles in protein-coding genes was analyzed according to previous published protocols [14]. For alleles located in protein-coding genes, the effects (synonymous or nonsynonymous) of mutations were determined by the Ensembl variant effect predictor tool [15]. After filtering out synonymous mutant alleles, the predicted effects of gout-associated nonsynonymous alleles on protein function were determined using the combination of the tools Polymorphism phenotyping (Polyphen), Sorting Intolerant From Tolerant (SIFT), and Predict the pathology of Mutations (PMUT) [16–18]. The predictions of each allele were stratified to form a 3-staged rating (Additional file 3) of the functional relevance. The scores from Polyphen, SIFT, and PMUT were combined by “averaging and rounding” to form a 3-staged rating (*neutral effect, **moderate evidence of pathogenicity, ***strong evidence of pathogenicity) for each allele (Fig. 1 Step 3a). For example, if the allele was rated as ** by Polyphen,** by SIFT, and *** by PMUT, the final score for this allele was ** (2 + 2 + 3 divided by 3 = 2.333, then rounded to 2).

### Functional annotation of alleles in transfer RNA (tRNA) genes

To annotate the impact of tRNA alleles, the Human Mitochondrial Genome Database (mtDB), Multiple Alignment using Fast Fourier Transform (MAFFT), and Mammalian Mitochondrial tRNA Genes (Mamit-tRNA) were used for the analysis of population genetics, evolutional conservation, and potential structural and functional alterations (Fig. 1, Step 3b), as described previously [19]. In the population genetics analysis step, we utilized mtDB to calculate the frequency of alleles in 2704 mitochondrial genomes [20]. In the evolutional conservation analysis step, orthologous sequences from human and 16 vertebrate species (Additional file 4) were aligned by MAFFT [21]. The conservation index (CI) was then estimated for identified alleles of interest. The CI was defined as the percentage of species from the 17 species (human and 16 vertebrate species) that had the wild-type nucleotide in a given position. In the final step, potential structural and functional alterations, the secondary structures of tRNA were retrieved from Mamit-tRNA for structural and functional evaluation and positions of nucleotides at tRNA were numbered according to conventional rules [22, 23]. Alleles were classified as potentially pathogenic alleles [19] if they met the criteria of (1) absent in the 104 non-gout controls and < 1% in the 2704 mitochondrial genomes, (2) evolutional conservation among 17 vertebrates (CI > 75%), and (3) potential structural and functional alterations.

### Validation of selected alleles detected by next-generation sequencing

To validate next-generation sequencing results, selected alleles (m.5628 T > C in *MT-TA* region and m.9957 T > C in *MT-CO3* region) were also analyzed by quantitative polymerase chain reaction, single nucleotide polymorphism (qPCR SNP) dual-fluorescent-labeled probe assays. qPCR SNP was performed using the StepOnePlus Real Time PCR platform (Applied Biosystems, USA). The assays were custom designed by Topgen Biotechnology (Topgen Biotech,Taiwan). The PCR primer sequences and probe sequences used are listed in Additional file 5. The PCR was performed with 2 × AceQ Probe Master Mix (Topgen Biotech, Taiwan). The cycling program for m.5628 T > C in *MT-TA* region included a pre-read stage at 60 °C for 30 s, a hold stage at 95 °C for 5 min, 40 cycles of PCR comprising denaturation at 95 °C for 3 s and annealing/extension at 64 °C for 40 s, and a post-read stage at 60 °C for 30 s. The amplification protocols for m.9957 T > C in *MT-CO3* region were as follows: a pre-read stage at 60 °C for 30 s, a hold stage at 95 °C for 5 min, a PCR stage at 95 °C for 3 s and 60 °C for 40 s for 40 cycles, and a post-read stage at 60 °C for 30 s. Allelic discrimination plots were analyzed by StepOnePlus SW v2.3.

### Heteroplasmic fraction (HF) calculation

Instead of having two copies of each autosome (chromosomes 1–22), human cells have 100’s to 1000’s of copies of mtDNA, and different copies of mtDNA may differ in DNA sequence at any base, a condition known as heteroplasmy. Thus there are only three discrete genotype states found in nuclear DNA, but pathogenic mitochondrial alleles can affect a varying proportion of mtDNA molecules - from 0% to 100%, and every possibility in between. Clinical manifestation therefore does not only depend on the specific mutation and the affected gene but also on the ratio of mutated to wild-type mtDNA, a condition called threshold effects [6]. As a result, the correlation between phenotype and genotype was considered in the context of HF, which is the proportion of mutant alleles at each site of the mitochondrial genome [6]. We utilized a previously published approach to determine the HF of each mutation [24]. In brief, we calculated the HF by dividing the number of mutant allele reads by the total number of reads at the same nucleotide position. For example, if 0.65% reads of m.9957 were reference allele (T) and 99.35% reads of m.9957 were mutant allele (C), then the HF of m.9957 would be 99.35%. The HF was considered further for the testing of correlation between phenotypes and genotypes (Fig. 1, Step 5).

### Statistical analyses

*P* values for continuous variables were calculated using Student’s *t* test or the Mann-Whitney U test when appropriate. Categorical variables were compared using the chi-square test or exact test. Given the exploratory nature of this study, *P* < 0.05 was considered statistically significant.

## Results

### Spectrum of mitochondrial genome mutant alleles

A total of 52 male patients with gout and 104 age-matched male controls were included in the study. The mean age of patients with gout was 51.60 ± 10.82 years, which was not significantly different to that of non-gout controls (51.61 ± 10.78 years, *P* = 0.996). Next-generation sequencing data were generated from all patients with a mean read depth across the mitochondrial genome of 3251.22 ± 964.63 (range 2027.23–5612.43). In the cohort of 52 patients with gout, 456 mutant alleles were identified compared to 640 mutant alleles from 104 age-matched non-gout controls. A total of 274 identified mutant alleles were shared by patients with gout and non-gout controls (Additional file 6). When we categorized mutant alleles according to their positions in the mitochondrial genomes, 45 mutant alleles in patients with gout were located in the *MT-ND5* region, followed-by the *MT-CYB* region (41 mutant alleles; 1 mutant allele was located in the overlapping *MT-ATP6* and *MT-ATP8* region) (Additional file 7). In the non-gout controls, the *MT-CYB* region contained 69 alleles, followed by the *MT-ND5* region (67 alleles; 3 mutant alleles were located in the overlapping *MT-ATP6* and *MT-ATP8* region) (Additional file 7). When we categorized mutant alleles according to their frequency in patients withg gout or non-gout controls, eight mutant alleles of the *MT-CYB* region in patients with gout were common alleles (frequency > 5%) (Additional file 8) [25]. Conversely, 33 mutant alleles of the *MT-CYB* region in patients with gout were low-frequency alleles (frequency < 5%) (Additional file 8) [25]. Similarly, there were seven common alleles of the *MT-CYB* region in non-gout controls, compared to 62 low-frequency alleles of the *MT-CYB* region in non-gout controls (Additional file 8). In general, there was a substantial excess of low-frequency alleles across various gene regions (Additional file 8).

### Mitochondrial mutant allele burdens in patients with gout and non-gout controls

When we compared the mean number of mutant alleles per individual across the mitochondrial genome, patients with gout and non-gout controls had comparable numbers of mutant alleles in respective regions (all *P* > 0.05) (Additional file 9). Thus, patients with gout and non-gout controls had similar mutant allele burdens.

### Mitochondrial alleles by allele groups

Next, we grouped mitochondrial alleles according to their distribution in patients with gout and non-gout controls and assessed their enrichment in patients with gout and non-gout controls (Fig. 1, Step 1) (group 1: mutant alleles shared by patients with gout and non-gout controls; group 2: mutant alleles found in patients with gout only; group 3: mutant alleles found in non-gout controls only). Most of the group-1 alleles, located in the *MT-CYB* and *MT-ND5* region (28 alleles), followed by the *MT-ND4* region (21 alleles) (Additional file 10). Most of the group-2 alleles were located in the *MT-CO1* and *MT-ND5* regions (17 alleles respectively) (Additional file 10). Most of the group-3 alleles were located in the *MT-CYB* region (41 alleles), followed by the *MT-ND5* region (39 alleles) (Additional file 10). There were 40 of 52 (76.92%) patients with gout with group-1 alleles in *MT-ATP6*, compared to 69 of 104 (66.35%) in non-gout controls, showing no evidence of enrichment (*P* = 0.175, chi-square test) (Table 1). Similarly, there was no significant enrichment of group-1 alleles over other gene regions in patients with gout or non-gout controls (all *P* > 0.05). *MT-ATP6* was significantly enriched for group-2 alleles in patients with gout (10 of 52 (19.23%) patients had group-2 alleles in *MT-ATP6*, *P* = 9.03 × 10^− 6^, exact test) (Table 1). *MT-ATP6* was significantly enriched for group-3 alleles in non-gout controls (25 of 104 (24.04%) controls, *P* = 1.14 × 10^− 4^, chi-square test) (Table 1). Various mitochondrial genes were largely significantly enriched for group-2 alleles or group-3 alleles in gout or non-gout controls. As *MT-ATP6* was significantly enriched for group-2 alleles (*P* = 9.03 × 10^− 6^) and group-3 alleles (*P* = 1.14 × 10^− 4^) (Table 1), positively associated and negatively associated alleles could be located within the same gene. Thus in the following analysis, we divided alleles in each gene to positively associated and negatively associated alleles to delineate the contribution of each to gout.

**Table 1:** Number of individuals with observed alleles in respective genes by allele group

Gene	Group 1			Group 2		Group 3	
	Gout *n* (%)	Non-gout *n* (%)	*P*	Gout *n* (%)	*P*	Non-gout *n* (%)	*P*
*MT-ATP6*	40 (76.92)	69 (66.35)	0.175	10 (19.23)	9.03 × 10^−6^	25 (24.04)	1.14 × 10^−4^
*MT-ATP8*	4 (7.69)	12 (11.54)	0.455	2 (3.85)	1.10 × 10^−1^	12 (11.54)	8.86 × 10^−3^
*MT-CO1*	26 (50.00)	48 (46.15)	0.650	14 (26.92)	5.56 × 10^−8^	39 (37.50)	3.41 × 10^−7^
*MT-CO2*	14 (26.92)	20 (19.23)	0.273	12 (23.08)	7.34 × 10^−7^	20 (19.23)	7.07 × 10^−4^
*MT-CO3*	42 (80.77)	78 (75.00)	0.420	6 (11.54)	1.12 × 10^−3^	17 (16.35)	2.01 × 10^− 3^
*MT-CYB*	52 (100.00)	104 (100.00)	1.000	10 (19.23)	9.03 × 10^−6^	40 (38.46)	2.15 × 10^−7^
*MT-ND1*	33 (63.46)	57 (54.81)	0.302	9 (17.31)	3.09 × 10^−5^	19 (18.27)	1.01 × 10^−3^
*MT-ND2*	37 (71.15)	69 (66.35)	0.544	8 (15.38)	1.04 × 10^−4^	22 (21.15)	3.46 × 10^− 4^
*MT-ND3*	46 (88.46)	88 (84.62)	0.515	2 (3.85)	1.10 × 10^−1^	17 (16.35)	2.01 × 10^−3^
*MT-ND4*	52 (100.00)	104 (100.00)	1.000	7 (13.46)	3.44 × 10^−2^	30 (28.85)	1.64 × 10^−5^
*MT-ND4L*	11 (21.15)	19 (18.27)	0.667	0 (0.00)	1.000	9 (8.65)	2.98× 10^−2^
*MT-ND5*	46 (88.46)	99 (95.19)	0.182	15 (28.85)	8.35 × 10^−9^	32 (30.77)	7.24 × 10^−6^
*MT-ND6*	31 (59.62)	57 (54.81)	0.568	9 (17.31)	3.09 × 10^−5^	18 (17.31)	1.42 × 10^−3^
*MT-RNR1*	35 (67.31)	74 (71.15)	0.622	4 (7.69)	1.14 × 10^−2^	20 (19.23)	7.07 × 10^−4^
*MT-RNR2*	52 (100.00)	104 (100.00)	1.000	5 (9.62)	3.60 × 10^−3^	25 (24.04)	1.14 × 10^−4^
*MT-TRNA* ^a^	26 (50.00)	54 (51.92)	0.821	18 (34.62)	1.78 × 10^−10^	28 (26.92)	3.62 × 10^−5^

For each gene, numbers and percentages of samples from patients with gout/non-gout with alleles of the respective gene that fall in each group are presented. Group 1, alleles shared by patients with gout and non-gout controls. Group 2, alleles in patients with gout only. Group 3, alleles found in non-gout controls only

^a^Please refer to Additional file 2 for more detailed information

### Association of mitochondrial genes with gout

Given the evidence for the presence of mitochondrial mutant alleles in gout and non-gout controls, we next identified genes associated with gout (Fig. 1, Step 2). Additionally, given the observation that synonymous mutant alleles had a neutral effect on protein function and disease susceptibility, we looked for nonsynonymous alleles among total alleles in the protein-coding genes (Additional file 11).

We utilized the gene burden ratio to classify alleles as positively associated alleles (gene burden ratio > 1) or negatively associated alleles otherwise. We then applied SKAT to calculate the contribution of nonsynonymous alleles and alleles over ribosomal RNA (rRNA) and tRNA genes in regards to gout susceptibility. Positively associated alleles were identified among eight genes (*MT-TA*, *MT-TC*, *MT-TH*, *MT-TQ*, *MT-TS2*, *MT-TT*, *MT-TW*, and *MT-CO3*) (Additional file 12). In contrast, negatively associated alleles among various genes were not observed to impact gout susceptibility (Additional file 12). Furthermore, genetic interactions between these positively associated loci were also observed (Additional file 13).

### Functional annotation of positively associated *MT-CO3* alleles

To clarify the functional relevance of *MT-CO3* alleles obtained from SKAT, PolyPhen, SIFT, and PMUT were applied to estimate the impact of these positively associated alleles (Fig. 1, Step 3a) [14]. This analysis suggests that four alleles showed moderate evidence of pathogenicity (**) (Table 2). Of the four potentially pathogenic alleles (m.9438G > A, m.9490C > T, m.9856 T > C, and m.9957 T > C), m.9438G > A, m.9856 T > C, and m.9957 T > C were associated with Leber’s hereditary optic neuropathy (LHON), left ventricular non-compaction cardiomyopathy (LVNC), mitochondrial encephalomyopathy, lactic acidosis, stroke-like episodes (MELAS), and non-arteritic ischemic optic neuropathy (NAION) [26–30]. Furthermore, all had a HF greater than 40% (Table 2) - the metabolic threshold shown in previous studies [31–33]. Altogether, this evidence suggests that these four potentially pathogenic *MT-CO3* alleles may be involved in the development of gout.

**Table 2:** Positively associated alleles of *MT-CO3* identified from SKAT analysis

Gene	Position	Mutation	Amino acid change	HF (%)	Impact^a^	Published disease association
*MT-CO3*	9438	G➔A	G78S	42.05	**	LHON [26]
*MT-CO3*	9490	C➔T	A95V	99.23	**	
*MT-CO3*	9755	G➔C	E183D	99.59	*	
*MT-CO3*	9856	T➔C	I217T	99.71	**	LVNC [27]
*MT-CO3* ^b^	9957	T➔C	F251 L	99.35 59.92	**	MELAS/NAION [28–30]

*Abbreviations*: *SKAT* Sequence Kernel Association Test, *HF* heteroplasmic fraction, *LHON* Leber’s hereditary optic neuropathy, *LVNC* left ventricular non-compaction cardiomyopathy, *MELAS* mitochondrial encephalomyopathy, lactic acidosis, and stroke-like episodes, *NAION* non-arteritic ischemic optic neuropathy

^a^Functional impact assessed by the combination of Polymorphism Phenotyping (Polyphen), Sorting Intolerant From Tolerant (SIFT), and Predict the Pathology of Mutation (PMUT) as described in “Methods”

^b^Two patients with gout had m.9957 T > C alleles

### Functional annotation of positively associated *MT-TA*, *MT-TC*, *MT-TH*, *MT-TQ*, *MT-TS2*, *MT-TT*, and *MT-TW* alleles

To clarify the relevance of positively associated alleles of tRNA genes identified from SKAT analysis, mtDB, MAFFT, and Mamit-tRNA were applied for the analysis of population genetics, evolutional conservation, and potential structural and functional alterations (Fig. 1, Step 3b) [19]. Of these alleles, m.5528 T > C, m.5557 T > C, m.5563G > A, m.5628 T > C, m.5783G > A, m.5814 T > C, m.12178C > T, m.12234A > G, m.12239C > T, m.12248A > G, m.15894G > A, and m.15940 T > C satisfied the criteria of being absent in the 104 non-gout controls and < 1% in the 2704 mitochondrial genomes (Table 3) [19]. With regard to evolutional conservation, only six alleles (m.5628 T > C, m.5783G > A, m.5814 T > C, m.12172A > G, m.12248A > G, and m.15894G > A) showed evolutional conservation (CI > 75%) (Table 3) [19].

**Table 3:** Positively associated alleles of *MT-TA*, *MT-TC*, *MT-TH*, *MT-TQ*, *MT-TS2*, *MT-TT*, and *MT-TW* identified from SKAT analysis

Gene	Position	Mutation	Location^a^	Nucleotide number^b^	HF (%)	Absent in control	Fre (%)^c^	CI (%)	Alteration^d^	Published disease association
*MT-TQ* ^e^	4386	T➔C	D-loop	15	99.8599.61	+	1.89	64.71		
*MT-TW*	5528	T➔C	D-loop	18	70.53	+	0.00	11.76		
*MT-TW*	5557	T➔C	V region	48	80.18	+	0.15	47.06		
*MT-TW*	5563	G➔A	T-loop	55	51.92	+	0.30	11.76		
*MT-TA*	5601	C➔T	T-loop	59	77.10	+	1.37	47.06		
*MT-TA*	5628	T➔C	AC stem	31	99.61	+	0.15	94.12	+	Hearing loss, hypertension, ophthalmoplegia and dysphagia [37–39]
*MT-TC*	5783	G➔A	T-stem	50	99.58	+	0.04	94.12	+	Encephalomyopathy, deafness, myopathy, cardiomyopathy, and renal failure [40, 41]
*MT-TC*	5814	T→C	D-stem	13	59.30	+	0.37	88.24	+	Encephalomyopathy [42]
*MT-TH*	12,172	A→G	AC loop	38	99.67	+	1.15	88.24	+	
*MT-TH*	12,178	C→T	V region	44	71.81	+	0.00	64.71	+	
*MT-TS2*	12,234	A→G	AC stem	42	55.90	+	0.30	58.82		
*MT-TS2*	12,239	C→T	V region	48	66.70	+	0.96	64.71		
*MT-TS2*	12,248	A→G	T-loop	57	99.59	+	0.11	100.00		
*MT-TT*	15,894	G→A	ACC stem	7	99.52	+	0.04	88.24	+	
*MT-TT*	15,940	T→C	T-loop	59	65.31	+	0.11	41.18		

*Abbreviations*: *SKAT* Sequence Kernel Association Test, *HF* heteroplasmic fraction, *CI* conservation index, *V* variable, *AC* anticodon, *ACC* acceptor

^a^The region of transfer RNA (tRNA) (acceptor stem, D-stem, D-loop, anticodon stem, anticodon loop, variable region, T-stem, T-loop) where alleles were located (Fig. 2) ^b^Nucleotide numbers represent the nucleotide positions according to the conventional tRNA numbering system, as described in “Methods”

^c^Allele frequency in 2704 mitochondrial genomes

^d^Potential structural and functional alterations

^e^Two patients with gout had m.4386 T > C alleles

**Fig. 2:**
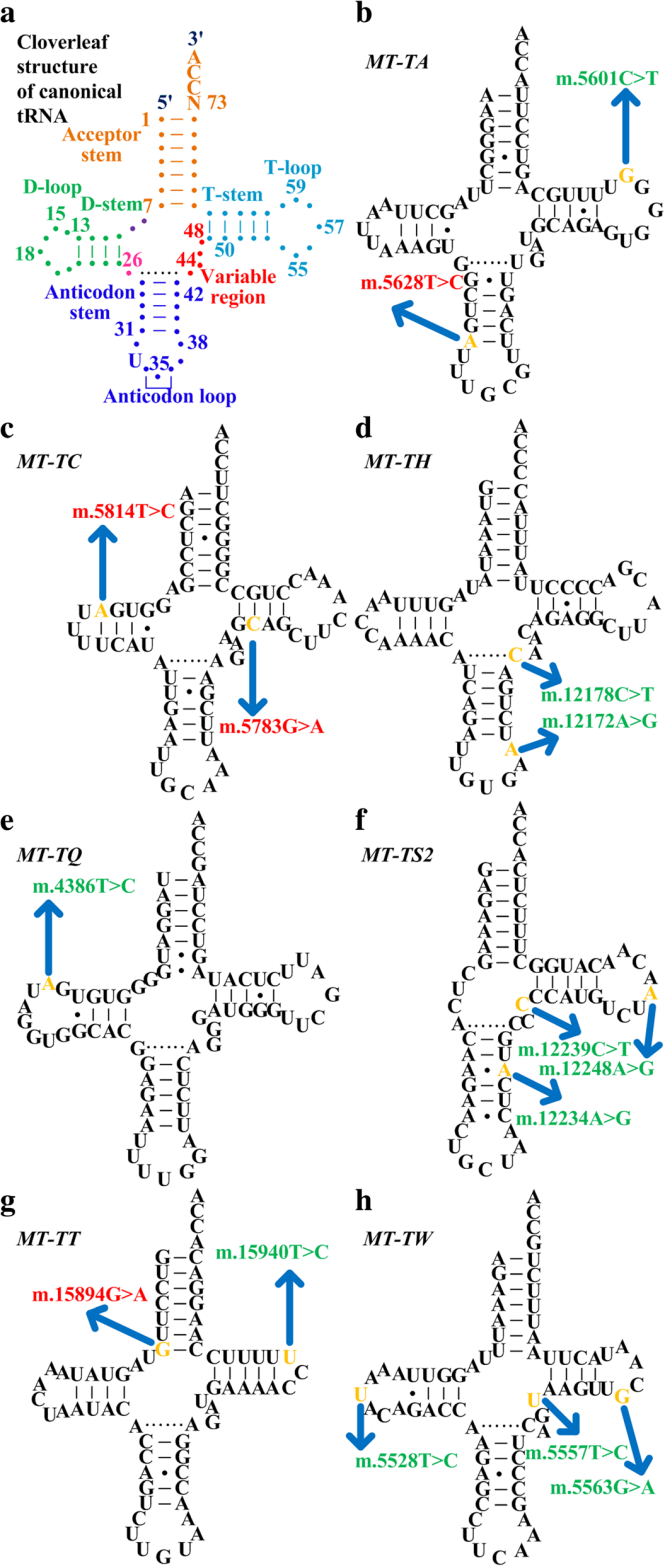
Mitochondrial positively associated alleles in gout-associated transfer RNA (tRNA) genes. **a** Cloverleaf structure of canonical tRNA. Various tRNA regions (acceptor stem, D-stem, D-loop, anticodon stem, anticodon loop, variable region, T-stem, T-loop) are shown. Numbers represent the nucleotide positions according to the conventional tRNA numbering system. Locations of tRNA positively associated alleles and cloverleaf structure of *MT-TA* (**b**), *MT-TC* (**c**), *MT-TH* (**d**), *MT-TQ* (**e**), *MT-TS2* (**f**), *MT-TT* (**g**), and *MT-TW* (**h**). Of all positively associated alleles in gout-associated tRNA genes, four alleles (m.5628 T > C, m.5783G > A, m.5814 T > C, and m.15894G > A) disrupting Watson-Crick base-pairing (A-U, G-C) were regarded as potentially pathogenic alleles. Tertiary interactions between nucleotides are indicated by dotted lines. Arrows indicate the position of the tRNA mutations. Red, potentially pathogenic alleles; green, nonpathogenic alleles

When we retrieved the tRNA structure from Mamit-tRNA, four alleles (m.5628 T > C in anticodon stem, m.5783G > A in T-stem, m.5814 T > C in D-stem, and m.15894G > A in acceptor stem) disrupted the classic Watson-Crick base-pairing (A-U, G-C) (Fig. 2b, c, g) [34]. Since Watson-Crick mispairing causes a defect in aminoacylation of tRNAs essential for protein synthesis [35], it was biologically plausible that these alleles have pathogenic potential in the development of gout. Although m.12172A > G was located in the anticodon loop (Fig. 2d) involved in codon-anticodon recognition, the allele frequency of m.12172A > G was 1.15% in the 2704 mitochondrial genome (Table 3), greater than the 1% of the pathogenicity criteria in our analysis [19]. Thus m.12172A > G was not regarded as a potentially pathogenic allele in this study. Similarly, m.12178C > T located at position 44 of the variable region (the nucleotide interacting with residue 26 (Fig. 2d) and participating in the formation of the tertiary structure [36]) was not regarded as potentially pathogenic given the CI was only 64.71% (Table 3), below the CI threshold of 75% [19].

Altogether, m.5628 T > C, combined with m.5783G > A, m.5814 T > C, and m.15894G > A, met the criteria of (1) being absent in the 104 non-gout controls and < 1% in the 2704 mitochondrial genomes, (2) evolutional conservation among 17 vertebrates (CI > 75%), and (3) potential structural and functional alterations (Table 3) [19]. Furthermore, all four alleles had a HF greater than 40% (Table 3) - the metabolic threshold shown in previous studies [31–33]. Three (m.5628 T > C, m.5783G > A, and m.5814 T > C corresponding to m.5814A > G in the coding light strand) of these four alleles have in previous studies been associated with hearing loss/deafness, hypertension, ophthalmoplegia and dysphagia, encephalomyopathy, myopathy, cardiomyopathy, and renal failure [37–42]. These findings suggest that these four potentially pathogenic alleles (m.5628 T > C, m.5783G > A, m.5814 T > C, and m.15894G > A) located in *MT-TA*, *MT-TC*, and *MT-TT* regions are possibly important in the pathogenesis of gout.

### Validation of selected alleles detected by next-generation sequencing

**Fig. 3:**
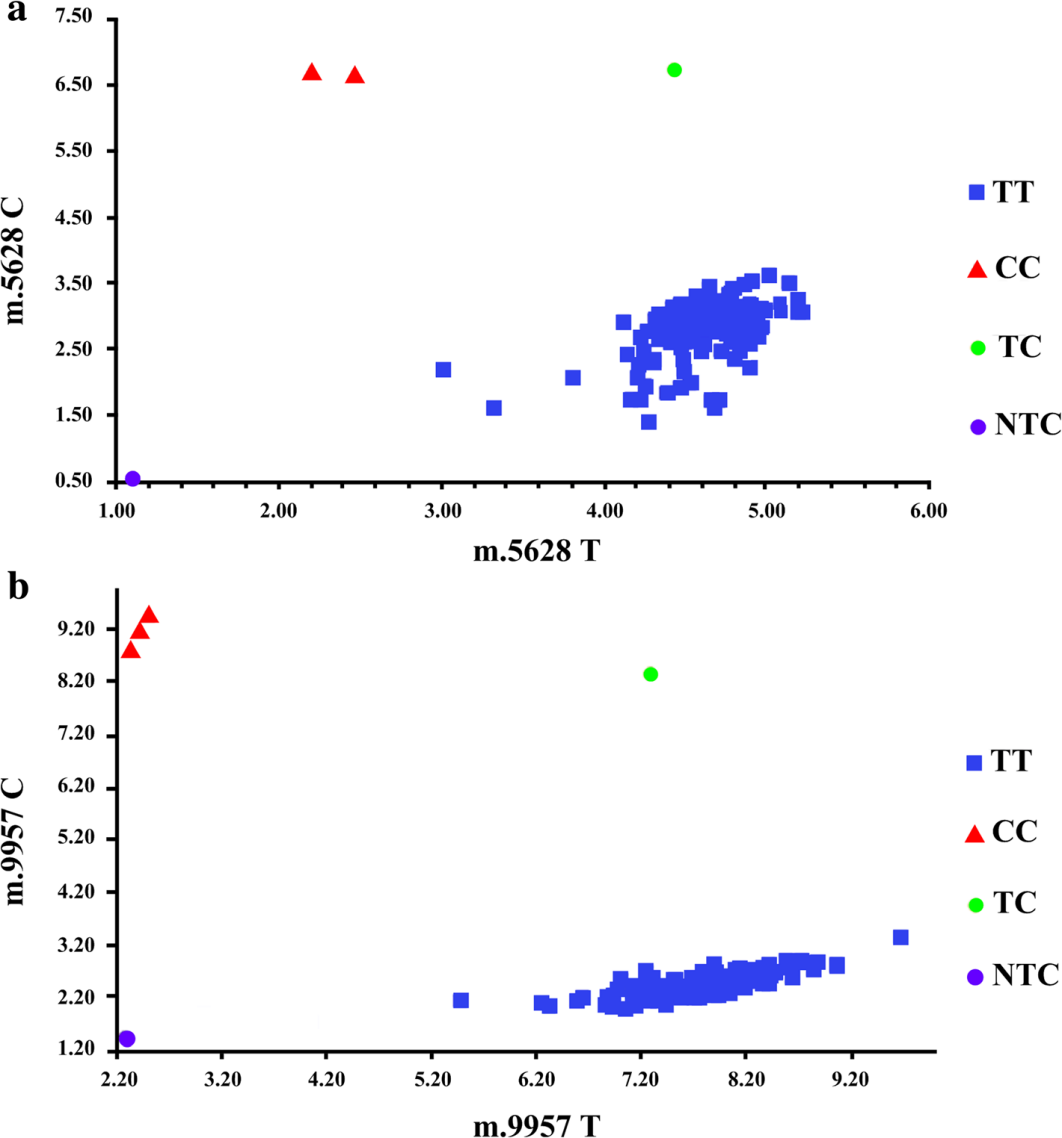
Analysis of m.5628 T > C and m.9957 T > C by quantitative polymerase chain reaction single nucleotide polymorphism. **a** m.5628 T > C genotyping showed one synthetic DNA construct carrying C allele (red triangle) and one mixture of synthetic DNA construct carrying C allele and synthetic DNA construct carrying T allele (green circle). Among samples from all patients with gout and non-gout controls, one sample carried C allele (red triangle) and all other samples carried T alleles (blue rectangle). **b** m.9957 T > C genotyping showed one synthetic DNA construct carrying C allele (red triangle) and one mixture of synthetic DNA construct carrying C allele and synthetic DNA construct carrying T allele (green circle). Among samples from all patients with gout and non-gout controls, two samples carried C allele (red triangle) and all other samples carried T alleles (blue rectangle)

To validate the accuracy of next-generation sequencing, we also analyzed selected alleles by qPCR SNP assays (Fig. 1, Step 4). We included two mitochondrial alleles for further validation: m.9957 T > C in *MT-CO3* region and m.5628 T > C in *MT-TA* region, which were both potentially pathogenic alleles in our functional annotation step and had established associations with disease. In the alleles examined by qPCR SNP assays, the results produced by qPCR SNP (Fig. 3) and next-generation sequencing were concordant in all samples.

### Association between mitochondrial potentially pathogenic alleles and patient phenotypes

*MT-CO3* encoded cytochrome c oxidase III, a subunit of respiratory complex IV. In addition to the role of mitochondria in gout [43], past studies also documented associations between mitochondrial alleles of respiratory complex IV genes and HDL [7]. Thus, we evaluated the functional impact of four potentially pathogenic *MT-CO3* alleles (m.9438G > A, m.9490C > T, m.9856 T > C, and m.9957 T > C) on HDL in patients (Fig. 1, Step 5). We observed higher HDL (*P* = 0.034) in patients with potentially pathogenic *MT-CO3* alleles than in patients without the corresponding alleles (Table 4). No relationship between the four potentially pathogenic *MT-CO3* alleles and other serum biochemical data was observed (Table 4). When we repeated the analysis for the four potentially pathogenic alleles in *MT-TA*, *MT-TC*, and *MT-TT* regions (m.5628 T > C, m.5783G > A, m.5814 T > C, and m.15894G > A), no difference was observed in serum biochemical data between patients with and without the four potentially pathogenic alleles in *MT-TA*, *MT-TC*, and *MT-TT* regions (Additional file 14).

**Table 4:** Associations between the presence of four potentially pathogenic *MT-CO3* alleles and clinical phenotypes in gout

Variables	Potentially pathogenic *MT-CO3* alleles		*P*
	+	–	
Number (percentage)	4 (7.69)	48 (92.31)	
Age (years; mean ± SD)	50.00 ± 12.99	51.73 ± 10.77	0.798
Uric acid (mg/dl; mean ± SD)	6.60 ± 2.07	7.71 ± 1.63	0.236
BMI (kg/m^2^; mean ± SD)	23.87 ± 2.09	26.70 ± 3.74	0.118
Diabetes mellitus (*n* (*%*))	1 (25.00)	3 (6.25)	0.281
Fasting glucose (mg/dl; mean ± SD)	98.50 ± 14.89	99.90 ± 19.31	0.973
Total cholesterol (mg/dl; mean ± SD)	192.20 ± 45.17	191.00 ± 39.92	0.693
HDL (mg/dl; mean ± SD)	61.00 ± 12.41	47.17 ± 12.60	0.034
LDL (mg/dl; mean ± SD)	119.30 ± 34.81	119.50 ± 38.81	0.918
Triglycerides (mg/dl; mean ± SD)	137.80 ± 86.89	159.10 ± 100.30	0.706

*Abbreviations*: *SD* standard deviation, *BMI* body mass index, *HDL* high-density lipoproteins, *LDL* low-density lipoproteins. *P*: Estimated by Mann-Whitney U or exact test

## Discussion

In this study, we identified a list of mitochondrial mutant alleles found in patients with gout and age-matched non-gout controls using next-generation sequencing. These results provided a comprehensive understanding of the mitochondrial genome landscape in patients with gout and showed an excess of low-frequency alleles in gout. When we compared the allele burden and assessed enrichment for certain mutant alleles in patients with gout and age-matched non-gout controls, the data suggested that patients with gout and age-matched non-gout controls have similar allele burdens but various mitochondrial genes were enriched for different sets of mutant alleles in patients with gout and non-gout controls. When we applied SKAT analysis, eight mitochondrial genes emerged as important contributors, with significant gene-gene interactions. After functional annotation, four alleles among *MT-CO3* and four alleles among *MT-TA*, *MT-TC*, and *MT-TT* with a HF higher than the metabolic threshold of pathogenicity were singled out through our analysis. Additionally, potentially pathogenic *MT-CO3* alleles had an impact on HDL, defining specific subpopulations within patients with gout.

This study identified the *MT-CO3*, *MT-TA*, *MT-TC*, and *MT-TT* region as the region containing potentially pathogenic alleles. *MT-CO3* encoded cytochrome c oxidase III, a subunit of respiratory complex IV (cytochrome c oxidase). This observation raised the possibility that respiratory complex IV dysfunction facilitates gouty attack. In line with this argument, past studies have documented the association between gout and multiple symmetric lipomatosis [44, 45] (also known as benign symmetric lipomatosis) [46], a disease characterized by complex IV dysfunction [47]. Furthermore, hydrogen sulfide, which inhibited complex IV, triggers inflammasome activation, the key step in gouty inflammation [3, 48, 49]. *MT-CO3* mutations increase reactive oxygen species (ROS) [50], which activates inflammasome, the link between urate crystals and gout [3, 51, 52]. Altogether, these findings suggest that *MT-CO3* alleles might mediate gout through respiratory complex IV dysfunction and subsequent ROS and inflammasome activation.

In addition to gout, potentially pathogenic *MT-CO3* alleles were associated with higher HDL in our cohort. The association between *MT-CO3* (encoding a subunit of complex IV) and HDL metabolism was further strengthened by the association between increased HDL and multiple symmetric lipomatosis, a disease characterized by complex IV dysfunction [46, 47]. Moreover, elevated blood levels of hydrogen sulfide, which inhibits complex IV, are positively correlated with HDL level [48, 53]. These findings are consistent with the relationship between *MT-CO3* alleles and HDL.

The *MT-TA*, *MT-TC*, and *MT-TT* genes encoded tRNAs participating in protein translation. Previous studies identified several mutations of *MT-TA* and *MT-TT* resulting in increased ROS production, which is linked to inflammasome activation and clinically presents as a gouty attack [3, 51, 52, 54, 55]. Thus, mitochondria *MT-TA*, *MT-TC*, and *MT-TT* alleles may facilitate gout attacks by enhancing ROS generation, a critical contributor of gouty attacks [56]. However, further mechanistic investigation is warranted.

Past studies suggest that mitochondria play an important role in gouty attacks [43]. Our findings support the results of those studies and provide a pathophysiological rationale for the therapeutic utility of mitochondria in gout. For example, resveratrol reduces mitochondrial ROS generation [57]. Past studies have also shown the potential of resveratrol in preventing gouty arthritis in animal models [58]. Therefore, one therapeutic strategy is to target mitochondria as a method of managing gout.

Regarding the limitations of this study, first, we utilized peripheral leukocytes instead of tissue cells as next-generation sequencing samples in this study. Since a central component of acute gouty inflammation involves leukocyte recruitment from blood into tissues [59], study of peripheral blood leukocytes could capture the mitochondrial genomic landscape of key (albeit not all) role players in gouty arthritis.

Next, there were concerns as to whether mutations detected in patients with gout actually provoked gout or should be considered an epiphenomenon caused by, for example, aging and ROS in gout [60, 61]. In response, the allele burdens across mitochondrial genes were comparable between patients with gout and age-matched non-gout controls. In addition, various mitochondrial genes were enriched for certain allele sets in patients with gout and age-matched non-gout controls. Moreover, the potentially pathogenic gout-associated alleles impacted HDL. Our findings suggest that random damage by aging and ROS cannot be the sole cause of mtDNA variations. Finally, in patients with gout, the majority of mutant alleles belonged to the category of low-frequency alleles (allele frequency < 5%) [25]. Although these alleles may have an effect on human phenotypes, large sequencing and genotyping projects will be required to uncover the full spectrum of these alleles and their effects on gout.

## Conclusion

In conclusion, this exploratory study suggests that mitochondrial alleles potentially play a role in the pathogenesis of gout and identify patient subgroups with distinct clinical phenotypes. Further validation and functional studies to clarify underlying mechanisms are recommended. Despite the mitochondrial genome encoding many proteins, mitochondria also depend on numerous nucleus-derived products in order to function properly. As an example, a previous GWAS implicated the *Glucokinase regulatory protein* (*GCKR*), which encodes protein associated with hepatic mitochondria, as one gout susceptibility gene [5, 62]. Furthermore, *ABC transporter subfamily G member 2* (*ABCG2*) regulates mitochondrial respiration, and is associated with gout [5, 63]. Thus, future studies on the interaction between the nuclear and mitochondrial genome may provide more insight into the role of mitochondria in gout. Furthermore, a systemic evaluation of mtDNA and nuclear mitochondrial genes to determine causality in gout-associated diseases employing a similar analysis framework and an investigation of epistatic interactions between the two genomes will reveal the extent to which mitochondrial defects play a causal role in human diseases.

## Additional files

Additional file 1: **Table S1.** Number of individuals by sequencing platforms in patients with gout and non-gout controls. (DOC 48 kb)

Additional file 2: **Table S2.** Gene symbols and names of total 37 genes in human mitochondria. (DOC 94 kb)

Additional file 3: **Table S3.** Stratification of the functional relevance for nonsynonymous mutations. (DOC 54 kb)

Additional file 4: **Table S4.** Species and accession numbers of mitochondrial genome sequences utilized in evolutional conservation analysis. (DOC 66 kb)

Additional file 5: **Table S5.** Polymerase chain reaction (PCR) primer and probe sequences for alleles studied. (DOC 94 kb)

Additional file 6: **Figure S1.** Number of mutant alleles in patients with gout and non-gout controls. (DOC 179 kb)

Additional file 7: **Table S6.** Number of alleles by gene region in patients with gout and non-gout controls. (DOC 79 kb)

Additional file 8: **Table S7.** Number of alleles by gene region and frequency in patients with gout and non-gout controls. (DOC 115 kb)

Additional file 9: **Table S8.** Mean allele counts per individual by gene region in patients with gout and non-gout controls. (DOC 103 kb)

Additional file 10: **Table S9.** Number of alleles by gene region and allele group in patients with gout and non-gout controls. (DOC 86 kb)

Additional file 11: **Table S10.** Number of nonsynonymous alleles by gene region and allele group in protein-coding genes. (DOC 49 kb)

Additional file 12: **Table S11.** Associations between mitochondrial genes and susceptibility to gout, stratified by positively associated alleles and negatively associated alleles. (DOC 86 kb)

Additional file 13: **Table S12.** Interaction analysis of genes with positively associated alleles. (DOC 54 kb)

Additional file 14: **Table S13.** Associations between the presence of four potentially pathogenic *MT-TA*, *MT-TC*, and *MT-TT* alleles and clinical phenotypes in gout. (DOC 61 kb)

## Abbreviations

ABCG2: ABC transporter subfamily G member 2
CI: Conservation index
EGC: Ethics and Governance Council
GCKR: Glucokinase regulatory protein
GWAS: Genome-wide association studies
HDL: High-density lipoprotein
HF: Heteroplasmic fraction
LHON: Leber’s hereditary optic neuropathy
LVNC: Left ventricular non-compaction cardiomyopathy
MAFFT: Multiple Alignment using Fast Fourier Transform
Mamit-tRNA: Mammalian Mitochondrial tRNA Genes
MELAS: Mitochondrial encephalomyopathy, lactic acidosis, and stroke-like episodes
MSU: Monosodium urate
mtDB: Human Mitochondrial Genome Database
mtDNA: Mitochondrial DNA
NAION: Non-arteritic ischemic optic neuropathy
Polyphen: Polymorphism Phenotyping
PMUT: Predict the Pathology of Mutations
qPCR: Quantitative polymerase chain reaction
ROS: Reactive oxygen species
rRNA: Ribosomal RNA
SIFT: Sorting intolerant from tolerant
SKAT: Sequence Kernel Association Test
SNP: Single nucleotide polymorphism
tRNA: Transfer RNA
TWB: Taiwan Biobank
