# Supplementary material for: Next-generation sequencing profiling of mitochondrial genomes in gout
Source: Arthritis Res Ther. 2018 Jul 6;20:137. doi: 10.1186/s13075-018-1637-5 (PMC6034246; doi:10.1186/s13075-018-1637-5)
Supplement: Supplementary file 1 — Table S1. Number of individuals by sequencing platforms in patients with gout and non-gout controls. (DOC 48 kb) [file 13075_2018_1637_MOESM1_ESM.doc]

**Table S1.** **Number of individuals by sequencing platforms in gout and non-gout controls.**

| Platform | Gout  n (%) | Non-gout  n (%) | *P* |
| --- | --- | --- | --- |
| Illumina Hiseq | 25 (48.08) | 50 (48.08) | 1.000 |
| Ion Proton | 27 (51.92) | 54 (51.92) |  |
