# Supplementary material for: Next-generation sequencing profiling of mitochondrial genomes in gout
Source: Arthritis Res Ther. 2018 Jul 6;20:137. doi: 10.1186/s13075-018-1637-5 (PMC6034246; doi:10.1186/s13075-018-1637-5)
Supplement: Supplementary file 2 — Table S2. Gene symbols and names of total 37 genes in human mitochondria. (DOC 94 kb) [file 13075_2018_1637_MOESM2_ESM.doc]

**Table S2. Gene symbols and names of total 37 genes in human mitochondria.**

| Symbols | Gene names |
| --- | --- |
| *MT-TA* | mitochondrially encoded tRNA alanine |
| *MT-TC* | mitochondrially encoded tRNA cysteine |
| *MT-TD* | mitochondrially encoded tRNA aspartic acid |
| *MT-TE* | mitochondrially encoded tRNA glutamic acid |
| *MT-TF* | mitochondrially encoded tRNA phenylalanine |
| *MT-TG* | mitochondrially encoded tRNA glycine |
| *MT-TH* | mitochondrially encoded tRNA histidine |
| *MT-TI* | mitochondrially encoded tRNA isoleucine |
| *MT-TK* | mitochondrially encoded tRNA lysine |
| *MT-TL1* | mitochondrially encoded tRNA leucine 1 |
| *MT-TL2* | mitochondrially encoded tRNA leucine 2 |
| *MT-TM* | mitochondrially encoded tRNA methionine |
| *MT-TN* | mitochondrially encoded tRNA asparagine |
| *MT-TP* | mitochondrially encoded tRNA proline |
| *MT-TQ* | mitochondrially encoded tRNA glutamine |
| *MT-TR* | mitochondrially encoded tRNA arginine |
| *MT-TS1* | mitochondrially encoded tRNA serine 1 |
| *MT-TS2* | mitochondrially encoded tRNA serine 2 |
| *MT-TT* | mitochondrially encoded tRNA threonine |
| *MT-TV* | mitochondrially encoded tRNA valine |
| *MT-TW* | mitochondrially encoded tRNA tryptophan |
| *MT-TY* | mitochondrially encoded tRNA tyrosine |
| *MT-TRNA* | *MT-TA, MT-TC, MT-TD, MT-TE, MT-TF, MT-TG, MT-TH, MT-TI, MT-TK, MT-TL1, MT-TL2, MT-TM, MT-TN, MT-TP, MT-TQ, MT-TR, MT-TS1, MT-TS2, MT-TT, MT-TV, MT-TW,* and *MT-TY* |
| *MT-RNR1* | mitochondrially encoded 12S RNA |
| *MT-RNR2* | mitochondrially encoded 16S RNA |
| *MT-ND1* | mitochondrially encoded NADH dehydrogenase 1 |
| *MT-ND2* | mitochondrially encoded NADH dehydrogenase 2 |
| *MT-ND3* | mitochondrially encoded NADH dehydrogenase 3 |
| *MT-ND4* | mitochondrially encoded NADH dehydrogenase 4 |
| *MT-ND4L* | mitochondrially encoded NADH 4L dehydrogenase |
| *MT-ND5* | mitochondrially encoded NADH dehydrogenase 5 |
| *MT-ND6* | mitochondrially encoded NADH dehydrogenase 6 |
| *MT-CYB* | mitochondrially encoded cytochrome b |
| *MT-CO1* | mitochondrially encoded cytochrome c oxidase I |
| *MT-CO2* | mitochondrially encoded cytochrome c oxidase II |
| *MT-CO3* | mitochondrially encoded cytochrome c oxidase III |
| *MT-ATP6* | mitochondrially encoded ATP synthase 6 |
| *MT-ATP8* | mitochondrially encoded ATP synthase 8 |
| *Noncoding* | Noncoding region |
