# Supplementary material for: Next-generation sequencing profiling of mitochondrial genomes in gout
Source: Arthritis Res Ther. 2018 Jul 6;20:137. doi: 10.1186/s13075-018-1637-5 (PMC6034246; doi:10.1186/s13075-018-1637-5)
Supplement: Supplementary file 3 — Table S3. Stratification of the functional relevance for nonsynonymous mutations. (DOC 54 kb) [file 13075_2018_1637_MOESM3_ESM.doc]

**Table S3.** **Stratification of the functional relevance for nonsynonymous mutations.**

| Rating | Polyphen | SIFT | PMUT |
| --- | --- | --- | --- |
| *** | Benign | Tolerated | Pathological, reliability score 0-3 |
| **** | Possibly damaging | Affects protein function (with low confidence) | Pathological, reliability score 4-6 |
| ***** | Probably damaging | Affects protein function | Pathological, reliability score 7-9 |

Abbreviations: SIFT = Sorting Intolerant From Tolerant, PMUT= Predict the pathology of MUTation.
