# Supplementary material for: Next-generation sequencing profiling of mitochondrial genomes in gout
Source: Arthritis Res Ther. 2018 Jul 6;20:137. doi: 10.1186/s13075-018-1637-5 (PMC6034246; doi:10.1186/s13075-018-1637-5)
Supplement: Supplementary file 4 — Table S4. Species and accession numbers of mitochondrial genome sequences utilized in evolutional conservation analysis. (DOC 66 kb) [file 13075_2018_1637_MOESM4_ESM.doc]

**Table S4.** **Species and accession numbers of mitochondrial genome sequences utilized in evolutional conservation analysis.**

| Species | GenBank accession number |
| --- | --- |
| *Bos taurus* | HM045018 |
| *Cebus albifrons* | NC_002763 |
| *Gorilla gorilla* | NC_011120 |
| *Hylobates lar* | NC_002082 |
| *Lemur catta* | NC_004025 |
| *Macaca mulatta* | NC_005943 |
| *Macaca sylvanus* | NC_002764 |
| *Mus musculus* | NC_006914.1 |
| *Nycticebus coucang* | NC_002765 |
| *Pan paniscus* | NC_001644 |
| *Pan troglodytes* | NC_001643 |
| *Papio hamadryas* | NC_001992 |
| *Pongo pygmaeus* | NC_001646 |
| *Pongo abelii* | NC_002083 |
| *Tarsius bancanus* | NC_002811 |
| *Xenopus laevis* | NC_001573.1 |
