# Supplementary material for: Next-generation sequencing profiling of mitochondrial genomes in gout
Source: Arthritis Res Ther. 2018 Jul 6;20:137. doi: 10.1186/s13075-018-1637-5 (PMC6034246; doi:10.1186/s13075-018-1637-5)
Supplement: Supplementary file 5 — Table S5. Polymerase chain reaction (PCR) primer and probe sequences for alleles studied. (DOC 94 kb) [file 13075_2018_1637_MOESM5_ESM.doc]

**Table S5. Polymerase chain reaction (PCR) primer and probe sequences for alleles studied.**

| Genes | Position | Allele 1^a^ | Allele 2^b^ | Allele 1 (VIC) probe | Allele 2 (FAM) probe | PCR forward primer | PCR reverse primer |
| --- | --- | --- | --- | --- | --- | --- | --- |
| *MT-TA* | 5628 | T | C | VIC-AGTGGCTG**A**TTTG-NFQ-MGB | FAM-AGTGGCTG**G**TTTG-NFQ-MGB | GCCCTCAGTAAGTTGCAATA CTTAATT | CCATTGGTCTAGTAAGGGCT TAGC |
| *MT-CO3* | 9957 | T | C | VIC-ACATACAGAA**A**TAGTC-NFQ-MGB | FAM-ACATACAGAA**G**TAGTC-NFQ-MGB | TTTACATCCAAACATCACTTTGGC | AAAGAGTAAGACCCTCATCAATAGATGG |

Abbreviations: VIC = 2'-chloro-7'phenyl-1,4-dichloro-6-carboxy-fluorescein, FAM = carboxyfluorescein, NFQ = nonfluorescent quencher, MGB = minor groove binder. Allele site: bold and underlined. ^a^Reference allele. ^b^Mutant allele.
