# Supplementary material for: Next-generation sequencing profiling of mitochondrial genomes in gout
Source: Arthritis Res Ther. 2018 Jul 6;20:137. doi: 10.1186/s13075-018-1637-5 (PMC6034246; doi:10.1186/s13075-018-1637-5)
Supplement: Supplementary file 6 — Figure S1. Number of mutant alleles in patients with gout and non-gout controls. (DOC 179 kb) [file 13075_2018_1637_MOESM6_ESM.doc]

**
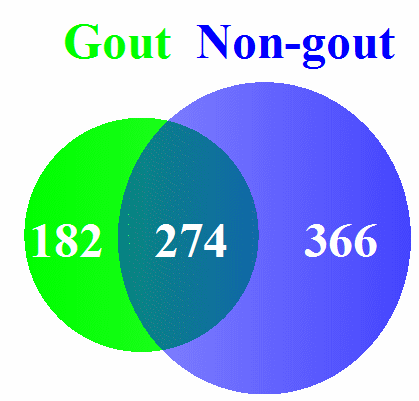
**

**Figure S1. Number of mutant alleles in gout patients and non-gout controls.** There were 182 alleles found in gout patients only, and 366 alleles found in non-gout controls only. 274 alleles were found in both gout patients and non-gout controls. Green: gout patients; blue: non-gout controls.
