# Supplementary material for: Next-generation sequencing profiling of mitochondrial genomes in gout
Source: Arthritis Res Ther. 2018 Jul 6;20:137. doi: 10.1186/s13075-018-1637-5 (PMC6034246; doi:10.1186/s13075-018-1637-5)
Supplement: Supplementary file 7 — Table S6. Number of alleles by gene region in patients with gout and non-gout controls. (DOC 79 kb) [file 13075_2018_1637_MOESM7_ESM.doc]

**Table S6. Number of alleles by gene region in gout and non-gout controls.**

| Gene | Gout | Non-gout |
| --- | --- | --- |
| *MT-ATP6* | 21 | 28 |
| *MT-ATP8* | 4 | 10 |
| *MT-CO1* | 28 | 42 |
| *MT-CO2* | 17 | 19 |
| *MT-CO3* | 18 | 25 |
| *MT-CYB* | 41 | 69 |
| *MT-ND1* | 29 | 35 |
| *MT-ND2* | 30 | 39 |
| *MT-ND3* | 7 | 16 |
| *MT-ND4* | 29 | 43 |
| *MT-ND4L* | 5 | 12 |
| *MT-ND5* | 45 | 67 |
| *MT-ND6* | 15 | 18 |
| *MT-RNR1* | 17 | 29 |
| *MT-RNR2* | 12 | 23 |
| *MT-TRNA*^a^ | 24 | 32 |
| *Noncoding*^a^ | 115 | 136 |

^a^Please refer to Additional file 2 for more detailed information.
