# Supplementary material for: Next-generation sequencing profiling of mitochondrial genomes in gout
Source: Arthritis Res Ther. 2018 Jul 6;20:137. doi: 10.1186/s13075-018-1637-5 (PMC6034246; doi:10.1186/s13075-018-1637-5)
Supplement: Supplementary file 8 — Table S7. Number of alleles by gene region and frequency in patients with gout and non-gout controls. (DOC 115 kb) [file 13075_2018_1637_MOESM8_ESM.doc]

**Table S7.** **Number of alleles by gene region and frequency in gout and non-gout controls.**

|  |  | Gout | |  | Non-gout | |
| --- | --- | --- | --- | --- | --- | --- |
| Frequency |  | >5% | <5% |  | >5% | <5% |
| *MT-ATP6* |  | 3 | 18 |  | 5 | 23 |
| *MT-ATP8* |  | 1 | 3 |  | 1 | 9 |
| *MT-CO1* |  | 4 | 24 |  | 5 | 37 |
| *MT-CO2* |  | 3 | 14 |  | 2 | 17 |
| *MT-CO3* |  | 3 | 15 |  | 5 | 20 |
| *MT-CYB* |  | 8 | 33 |  | 7 | 62 |
| *MT-ND1* |  | 7 | 22 |  | 5 | 30 |
| *MT-ND2* |  | 11 | 19 |  | 8 | 31 |
| *MT-ND3* |  | 3 | 4 |  | 3 | 13 |
| *MT-ND4* |  | 8 | 21 |  | 7 | 36 |
| *MT-ND4L* |  | 3 | 2 |  | 1 | 11 |
| *MT-ND5* |  | 14 | 31 |  | 9 | 58 |
| *MT-ND6* |  | 4 | 11 |  | 3 | 15 |
| *MT-RNR1* |  | 5 | 12 |  | 5 | 24 |
| *MT-RNR2* |  | 5 | 7 |  | 6 | 17 |
| *MT-TRNA*^a^ |  | 1 | 23 |  | 1 | 31 |
| *Noncoding*^a^ |  | 40 | 75 |  | 31 | 105 |

^a^Please refer to Additional file 2 for more detailed information.
