# Supplementary material for: Next-generation sequencing profiling of mitochondrial genomes in gout
Source: Arthritis Res Ther. 2018 Jul 6;20:137. doi: 10.1186/s13075-018-1637-5 (PMC6034246; doi:10.1186/s13075-018-1637-5)
Supplement: Supplementary file 9 — Table S8. Mean allele counts per individual by gene region in patients with gout and non-gout controls. (DOC 103 kb) [file 13075_2018_1637_MOESM9_ESM.doc]

**Table S8. Mean allele counts per individual by gene region in gout and non-gout controls**.

| Gene | Gout | Non-gout | *P* |
| --- | --- | --- | --- |
| *MT-ATP6* | 1.1923 ± 0.8411 | 1.2788 ± 1.0377 | 0.603 |
| *MT-ATP8* | 0.1346 ± 0.3975 | 0.2404 ± 0.4514 | 0.154 |
| *MT-CO1* | 1.0962 ± 0.9343 | 1.1538 ± 1.1040 | 0.747 |
| *MT-CO2* | 0.6154 ± 0.8438 | 0.5192 ± 0.7630 | 0.475 |
| *MT-CO3* | 1.6923 ± 1.0392 | 1.4135 ± 1.0203 | 0.112 |
| *MT-CYB* | 3.8846 ± 1.5423 | 3.9712 ± 1.6806 | 0.756 |
| *MT-ND1* | 1.3654 ± 1.1552 | 1.1635 ± 1.1070 | 0.292 |
| *MT-ND2* | 1.5000 ± 1.0756 | 1.3365 ± 1.0298 | 0.359 |
| *MT-ND3* | 1.2885 ± 0.6668 | 1.2885 ± 0.8205 | 1.000 |
| *MT-ND4* | 3.4231 ± 1.4996 | 2.9808 ± 1.5327 | 0.089 |
| *MT-ND4L* | 0.2885 ± 0.6051 | 0.3269 ± 0.5984 | 0.707 |
| *MT-ND5* | 2.6346 ± 1.5085 | 2.4904 ± 1.7180 | 0.608 |
| *MT-ND6* | 1.3654 ± 1.1207 | 1.3654 ± 1.2232 | 1.000 |
| *MT-RNR1* | 1.5577 ± 1.2432 | 1.8846 ± 1.4163 | 0.159 |
| *MT-RNR2* | 1.7692 ± 0.7307 | 2.0192 ± 0.8700 | 0.077 |
| *MT-TRNA*^a^ | 0.9423 ± 0.8264 | 0.8846 ± 0.8162 | 0.679 |
| *Noncoding*^a^ | 10.6154 ± 2.466 | 10.5000 ± 2.481 | 0.784 |

^a^Please refer to Additional file 2 for more detailed information.
