# Supplementary material for: Next-generation sequencing profiling of mitochondrial genomes in gout
Source: Arthritis Res Ther. 2018 Jul 6;20:137. doi: 10.1186/s13075-018-1637-5 (PMC6034246; doi:10.1186/s13075-018-1637-5)
Supplement: Supplementary file 10 — Table S9. Number of alleles by gene region and allele group in patients with gout and non-gout controls. (DOC 86 kb) [file 13075_2018_1637_MOESM10_ESM.doc]

**Table S9.** **Number of alleles by gene region and allele group in gout and non-gout controls.**

| Gene | Group 1 | Group 2 | Group 3 |
| --- | --- | --- | --- |
| *MT-ATP6* | 9 | 12 | 19 |
| *MT-ATP8* | 2 | 2 | 8 |
| *MT-CO1* | 11 | 17 | 31 |
| *MT-CO2* | 6 | 11 | 13 |
| *MT-CO3* | 12 | 6 | 13 |
| *MT-CYB* | 28 | 13 | 41 |
| *MT-ND1* | 18 | 11 | 17 |
| *MT-ND2* | 20 | 10 | 19 |
| *MT-ND3* | 5 | 2 | 11 |
| *MT-ND4* | 21 | 8 | 22 |
| *MT-ND4L* | 5 | 0 | 7 |
| *MT-ND5* | 28 | 17 | 39 |
| *MT-ND6* | 7 | 8 | 11 |
| *MT-RNR1* | 13 | 4 | 16 |
| *MT-RNR2* | 7 | 5 | 16 |
| *MT-TRNA*^a^ | 4 | 20 | 28 |

Group 1: shared by gout patients and non-gout controls; Group 2: found in gout patients only; Group 3: found in non-gout controls only. ^a^Please refer to Additional file 2 for more detailed information.
