# Supplementary material for: Next-generation sequencing profiling of mitochondrial genomes in gout
Source: Arthritis Res Ther. 2018 Jul 6;20:137. doi: 10.1186/s13075-018-1637-5 (PMC6034246; doi:10.1186/s13075-018-1637-5)
Supplement: Supplementary file 11 — Table S10. Number of nonsynonymous alleles by gene region and allele group in protein-coding genes. (DOC 49 kb) [file 13075_2018_1637_MOESM11_ESM.doc]

**Table S10.** **Number of nonsynonymous alleles by gene region and allele group in protein-coding genes.**

| Gene | Group 1 | Group 2 | Group 3 |
| --- | --- | --- | --- |
| *MT-ATP6* | 6 | 6 | 8 |
| *MT-ATP8^a^* | 1 | 1 | 3 |
| *MT-CO1* | 2 | 4 | 8 |
| *MT-CO2* | 1 | 2 | 2 |
| *MT-CO3* | 1 | 4 | 5 |
| *MT-CYB* | 13 | 6 | 12 |
| *MT-ND1* | 5 | 5 | 5 |
| *MT-ND2* | 6 | 1 | 6 |
| *MT-ND3* | 2 | 1 | 2 |
| *MT-ND4* | 3 | 3 | 3 |
| *MT-ND4L* | 1 | 0 | 3 |
| *MT-ND5* | 13 | 6 | 6 |
| *MT-ND6* | 2 | 2 | 1 |

Group 1: shared by gout patients and non-gout controls; Group 2: found in gout patients only; Group 3: found in non-gout controls only. ^a^One Group 2 allele located in the overlapping region of *MT-ATP6* and *MT-ATP8* caused nonsynonymous mutations for both *MT-ATP6* and *MT-ATP8*.
