# Supplementary material for: Next-generation sequencing profiling of mitochondrial genomes in gout
Source: Arthritis Res Ther. 2018 Jul 6;20:137. doi: 10.1186/s13075-018-1637-5 (PMC6034246; doi:10.1186/s13075-018-1637-5)
Supplement: Supplementary file 12 — Table S11. Associations between mitochondrial genes and susceptibility to gout, stratified by positively associated alleles and negatively associated alleles. (DOC 86 kb) [file 13075_2018_1637_MOESM12_ESM.doc]

**Table S11.** **Associations of mitochondria genes with gout susceptibility, stratified by positively associated alleles and negatively associated alleles.**

| Gene | Positively associated alleles^a^ |  | Negatively associated alleles^b^ |
| --- | --- | --- | --- |
| *MT-TA R1* | 0.024* |  | - |
| *MT-TC R1* | 0.024* |  | 1.000* |
| *MT-TD* | - |  | 0.754* |
| *MT-TE R1* | 0.080* |  | 0.619* |
| *MT-TF* | - |  | 0.754* |
| *MT-TG* | - |  | 0.754* |
| *MT-TH R1* | 0.024* |  | 0.884* |
| *MT-TK R1* | 0.080* |  | 0.754* |
| *MT-TL1* | - |  | 0.754* |
| *MT-TL2 R* | 0.467* |  | 0.754* |
| *MT-TM* | - |  | 0.402* |
| *MT-TP* | - |  | 0.754* |
| *MT-TQ R1* | 0.020* |  | 0.835* |
| *MT-TR R1* | 0.080* |  | 0.288* |
| *MT-TS1 1* | 0.080* |  | - |
| *MT-TS2 1* | 0.008* |  | 0.754* |
| *MT-TT R1* | 0.024* |  | 0.838* |
| *MT-TV* | - |  | 0.405* |
| *MT-TWR1* | 0.008* |  | 0.754* |
| *MT-RNR1* | 0.784* |  | 0.322* |
| *MT-RNR2* | 0.122* |  | 0.191* |
| *MT-ND1R* | 0.325* |  | 0.900* |
| *MT-ND2 R* | 0.077* |  | 0.215* |
| *MT-ND3 R* | 0.475* |  | 0.835* |
| *MT-ND4 R* | 0.383* |  | 0.839* |
| *MT-ND4L* | - |  | 0.548* |
| *MT-ND5R* | 0.155* |  | 0.739* |
| *MT-ND6R* | 0.555* |  | 0.398* |
| *MT-CYB6* | 0.199* |  | 0.999* |
| *MT-CO1R* | 0.126* |  | 0.835* |
| *MT-CO2R* | 0.190* |  | 0.835* |
| *MT-CO3R* | 0.030* |  | 0.499* |
| *MT-ATP6* | 0.141* |  | 0.229* |
| *MT-ATP8* | 0.080* |  | 1.000* |

The data were presented as *P* values obtained by SKAT. For example, positively associated alleles in *MT-TC* were associated with gout (*P*=0.024) but negatively associated alleles in *MT-TC* were not (*P*=1.000). ^a^No positively associated alleles were located in *MT-TD, MT-TF, MT-TG, MT-TI, MT-TL1, MT-TM, MT-TN, MT-TP, MT-TV, MT-TY, MT-ND4L.*^b^No negatively associated alleles were located in *MT-TA, MT-TI, MT-TN, MT-TS1, MT-TY.* *: *P*<0.05.
