# Supplementary material for: Next-generation sequencing profiling of mitochondrial genomes in gout
Source: Arthritis Res Ther. 2018 Jul 6;20:137. doi: 10.1186/s13075-018-1637-5 (PMC6034246; doi:10.1186/s13075-018-1637-5)
Supplement: Supplementary file 13 — Table S12. Interaction analysis of genes with positively associated alleles. (DOC 54 kb) [file 13075_2018_1637_MOESM13_ESM.doc]

**Table S12. Interaction analysis of genes with positively associated alleles**.

|  | protein-coding |  | tRNA genes | | | | | |
| --- | --- | --- | --- | --- | --- | --- | --- | --- |
|  | genes |  |  |  |  |  |  |  |
|  | *MT-CO3* |  | *MT-TA* | *MT-TC* | *MT-TH* | *MT-TQ* | *MT-TS2* | *MT-TT* |
| tRNA genes | |  |  |  |  |  |  |  |
| *MT-TA3* | 0.014 |  |  |  |  |  |  |  |
| *MT-TC3* | 0.013 |  | 0.003 |  |  |  |  |  |
| *MT-TH3* | 0.013 |  | 0.003 | 0.003 |  |  |  |  |
| *MT-TQ3* | 0.004 |  | 0.007 | 0.007 | 0.007 |  |  |  |
| *MT-TS23* | 0.008 |  | 0.001 | 0.001 | 0.001 | 0.004 |  |  |
| *MT-TT3* | 0.014 |  | 0.003 | 0.003 | 0.003 | 0.013 | 0.001 |  |
| *MT-TW3* | 0.009 |  | 0.001 | 0.001 | 0.001 | 0.004 | 0.000 | 0.001 |

The data were presented as P values for departure from a multiplicative interaction model obtained by SKAT.
