# Supplementary material for: Next-generation sequencing profiling of mitochondrial genomes in gout
Source: Arthritis Res Ther. 2018 Jul 6;20:137. doi: 10.1186/s13075-018-1637-5 (PMC6034246; doi:10.1186/s13075-018-1637-5)
Supplement: Supplementary file 14 — Table S13. Associations between the presence of four potentially pathogenic MT-TA, MT-TC, and MT-TT alleles and clinical phenotypes in gout. (DOC 61 kb) [file 13075_2018_1637_MOESM14_ESM.doc]

**Table S13. Associations between the presence of four potentially pathogenic *MT-TA, MT-TC,* and *MT-TT* alleles and clinical phenotypes in gout.**

| Variables | Potentially pathogenic *MT-TA, MT-TC,* and *MT-TT* alleles | | | *P* | |
| --- | --- | --- | --- | --- | --- |
|  | + | - | |  |  |
| No. (n; %) | 4 (7.69) | 48 (92.31) | |  | |
| Age (years; mean ± SD) | 55.50 ± 12.87 | 51.27 ± 10.73 | | 0.439 | |
| Uric acid (mg/dl; mean ± SD) | 7.63 ± 2.05 | 7.63 ± 1.66 | | 0.731 | |
| BMI (kg/m^2^; mean ± SD) | 27.34 ± 6.03 | 26.41 ± 3.53 | | 0.823 | |
| Diabetes (n; %) | 0 (0.00) | 4 (8.33) | | 1.000 | |
| Fasting sugar (mg/dl; mean ± SD) | 94.00 ± 9.83 | | 100.27 ± 19.44 | | 0.770 |
| Total Cholesterol (mg/dl; mean ± SD) | 185.50 ± 26.94 | 191.56 ± 40.93 | | 0.757 | |
| HDL (mg/dl; mean ± SD) | 43.25 ± 5.56 | 48.65 ± 13.39 | | 0.492 | |
| LDL (mg/dl; mean ± SD) | 126.75 ± 27.42 | 118.92 ± 39.12 | | 0.693 | |
| Triglycerides (mg/dl; mean ± SD) | 89.00 ± 41.98 | 163.19 ± 100.18 | | 0.074 | |

Abbreviations: SD = standard deviation, BMI = body mass index, HDL = high-density lipoproteins, LDL = low-density lipoproteins. *P*: estimated by Mann-Whitney U or exact test.
